# Adult ADHD screening scores and hospitalization due to pedestrian injuries: a case-control study

**DOI:** 10.1186/s12888-020-02848-x

**Published:** 2020-09-10

**Authors:** Alireza Sadeghpour, Homayoun Sadeghi-Bazargani, Saber Ghaffari-fam, Shaker Salarilak, Mostafa Farahbakhsh, Robert Ekman, Amin Daemi

**Affiliations:** 1 Department of Orthopedics, Tabriz University of Medical Sciences, Tabriz, Iran; 2 Road Traffic Injury Research Center, Tabriz University of Medical Sciences, Central campus, Golshahr square, Elgoli Ave, Tabriz, 5167846185 Iran; 3 School of Nursing of Miyandoab, Urmia University of Medical Sciences, Urmia, Iran; 4 Department of Public Health, Islamic Azad University of Tabriz, Medical school, Tabriz, Iran; 5 Research Center of Psychiatry and Behavioral Sciences, Tabriz University of Medical Sciences, Tabriz, Iran; 6 Department of Architecture and Civil Engineering, Chalmers University of Technology, Gothenburg, Sweden; 7 Health Management and Economics Research Center, Iran University of Medical Sciences, Tehran, Iran

**Keywords:** Attention-deficit/hyperactivity disorder (ADHD), ADHD symptoms, Pedestrian, Road traffic accidents (RTAs), Distraction, Determinants, Case-control studies, Epidemiology, Iran

## Abstract

**Background:**

The aim of this study was to investigate the association between adult ADHD screening scores and hospitalization due to pedestrian injuries in a sample of Iranian pedestrians.

**Methods:**

Through a case-control study, a case population of 177 pedestrians injured by the vehicles in road traffic crashes were compared with 177 controls who lacked a record of intentional or unintentional injuries enrolled from various wards of Imam Reza University Hospital which is a specialty teaching hospital located in the same city with similar referral level. The cases and controls had an age range of 18–65 years and were matched on gender and age. ADHD symptom profile was assessed using the Persian Self-report Screening Version of the Conner’s Adult ADHD Rating Scales (CAARS-S:SV). The association of ADHD screening score and pedestrian injuries was investigated using multiple binary logistic regression to investigate the independent effect of ADHD index score on belonging to case group. Both crude and adjusted odds ratios were reported.

**Results:**

Men comprised 86.4% of the study subjects. The crude odds ratios for all the four ADHD subscales to be associated with pedestrian injuries were 1.05, 1.08, and 1.04 for the subscales A (attention deficit), B (hyperactivity/impulsiveness) and ADHD index respectively. However, the association for subscale A was not statistically significant with a borderline *p*-value. The final multivariate analysis showed that variables associated with pedestrian injuries in the road traffic crashes were ADHD Index score (OR = 1.06, 95% CI: 1.01–1.12); economic status (including household income and expenditure capacity); educational level and total walking time per 24 h.

**Conclusions:**

Adult ADHD screening score can predict pedestrian injuries leading to hospitalization independently from sex, age, economic status, educational level and pedestrian exposure to traffic environment (average walking time).

## Background

According to World Health Organization, 1.27 million fatal road traffic injuries and 20–50 million non-fatal injuries occur as a result of road traffic accidents occur annually with a higher burden in low-and middle-income countries (LMICs) [1]. Road traffic accidents are a major public health problem in Iran stated to be due to a diversity of reasons including the young population of the country which is associated with a greater degree of exposure to road traffic accidents; low prices for automobile fuel; lower vehicle safety designs [2, 3]. Pedestrians form a particularly vulnerable group for road traffic injuries, comprising nearly a quarter of all the road traffic fatalities [1].

### ADHD and traffic injuries

Risk factors for moderate to severe injuries from road traffic accidents are comprised of human, environmental, and vehicle-related factors. Psychological/psychiatric factors have always been the focus of interest in field of road traffic injuries/accidents (RTAs). Attention deficit hyperactivity disorder (ADHD) is an important clinical condition mainly affecting children but also relatively common among adults [4]. ADHD is specified as a familial disorder [5]. Longitudinal studies have estimated that approximately 65% of children with ADHD continue to show symptoms in adulthood [6]. The fundamental deficiency in ADHD is considered to be the failure to inhibit or delay behavioral responses [7]. ADHD has been shown to be associated with various unwanted outcomes like suicide, substance use, sleep problems, increased criminality, risky behaviors and injury [8]. It is also associated with lower economic status and functional impairments including lower levels of education and higher levels of unemployment.

Several studies including two recent reviews have been published showing an association between ADHD and various types of injuries both in childhood and adults. It has been shown that childhood ADHD increases the likelihood of injuries by 2.8 times and it has been shown that with ten units increment in the score of Hyperactive-Impulsive subscale of ADHD, leads to 73% increment in the chance of burn injuries [9]. ADHD has also been shown to increase the likelihood of falls and dental trauma [10, 11].

### Injury mechanisms in ADHD

Some characteristics of patients with ADHD could reasonably explain the association between ADHD and injuries. First of all, it could be the risk-taking behaviors that are much more prominent in ADHD. Risk-taking, which is a well-known predictor in road traffic injuries, could either increase the risks of other types of injuries such as falls or burns. Another feature is the attention deficit component of the disorder which is more prominently known risk factor for traffic injuries [10]. Other mechanism that explains the increased risk of accidents in ADHD is the executive dysfunction. The executive function (EF) is a top down process that regulates thoughts and behaviors of a person with control of sensory information and attention. The structure of this processes is in prefrontal cortex of brain. Working memory, response inhibition and set shifting are the three main functions of EF. In driving, high level cognitive processes are called EF that play supervisory or managerial role in complex behaviors [12, 13].

### Risky driving behaviors in ADHD

Various studies have focused specifically on assessing the association of ADHD with traffic-related injuries. Some have considered investigating the association between ADHD with respect to victim’s role in road traffic environment (drivers, riders, pedestrians and passengers). However, majority of the previous studies have been conducted on driving behavior or risk-of-crash among drivers with ADHD [14–20]. The general risk of crash in drivers with ADHD is well documented, primarily through observational studies and driving simulator studies, which has led to later clinical trials about efficacy of treatment on driving performance [21–25]. Vaa in a meta-analysis showed that relative risk of motor vehicle accidents in ADHD afflicted drivers were 1.36 in comparison to general population [26]. Fuermaier et al. in their review showed that ADHD patients, in comparison to general population, had higher number of injuries in motor vehicle crashes. They also reported that medications especially stimulants had improving effects on crash rate and outcomes [16]. Bron et al. in their cross sectional study showed that adult ADHD increased the odds of having three or more accidents in comparison to control group. They used a self-report questionnaire of driving behavior in 330 adults [17]. In another study, it was reported that every unit increase in ADHD severity score increased the chance of motor vehicles accidents by 5% [18]. Very few observational studies have also investigated the association of ADHD with riding behavior and risk of crash among motorcycle and bicycle riders [27–33] [14–20, 26, 28–30, 34, 35]. Efforts on increasing awareness of ADHD and screening of drivers for ADHD with subsequent evaluation and treatment are recommended for driver-related safety promotion [36] and it has also been shown that the hazard perception skills of drivers with ADHD can be improved using interventions such as computer based driver training even without pharmacologic treatment [37]. Pedestrians, although comprising a substantial proportion of road traffic fatalities, have been overlooked by researchers investigating role-specific association between adult ADHD and RTAs. The few available studies have focused on child or adolescent pedestrians. To the best of our knowledge, no previous study has been published on association of ADHD symptoms and risk of adult pedestrian injuries. There is also lack of evidence on patterns of the association of traffic injuries and ADHD with respect to the subtypes of ADHD. In two previous studies by our own team on association of ADHD and motorcycle injuries, controversial findings have been reached regarding the subtypes of ADHD [30, 33]. Moreover, there are some demographic features shown to be associated both with ADHD and injuries. Age, gender, educational level, smoking, marital status and economic status are among these factors [38–43].

### Current study

Therefore, any assessment of the effect of ADHD on injuries should control for these factors, either through matching or multivariate analysis which is not the case with all the available evidence. The aim of current study was to investigate the following alternative hypotheses:

1. Those adults with a higher score on ADHD index, using Conner’s ADHD screening tool, could have higher likelihood of being injured and hospitalized due to pedestrian injuries in a sample of Iranian pedestrians.
2. The potential association between pedestrian injuries and ADHD screening scores could vary with respect to the two types of attention deficit and hyperactivity/impulsiveness symptoms.

## Methods

### Study design and participants

This study employs a case-control design, and was conducted in two university hospitals of Tabriz University of Medical Sciences, located in in North-West of Iran. The study enrolled 177 pedestrians injured in road traffic accidents who were hospitalized during the years 2012–2013. Cases were patients receiving care from Shohada University Hospital located in Tabriz. The hospital is the regional trauma referral center in East Azerbaijan Province. Inclusion criteria for cases included people who had been hospitalized for 24 h or more in Shohada University Hospital due to pedestrian injuries in road traffic crashes, Patients were recruited from both genders. All patients lived in East Azerbaijan Province, and ranged 18–65 years of age. Exclusion criteria from the study included patients who were injured in other accidents, intentional injuries, patients who died before hospital admission, and those who were unable to respond due to severe injury. Of the 182 potential recruits to this study, five patients declined to participate.

For this study’s control group, 177 patients were enrolled from various wards of Imam Reza University Hospital which is a specialty teaching hospital located in the same city with similar referral level. This hospital serves as a referral center for the province, serving the same geographic population as Shohada Hospital for most admissions. Selecting controls was done according to the principals described by Wacholder et al. [44]. For instance, trying to enroll controls from a setting with similar referral level as cases increases the likelihood of having common source population (study base) or selecting controls from non-traumatic admissions to ensure independence of exposure from selection. The nonresponse, which was trivial in current study, is another source of selection bias in cases control studies. Information bias exists “when cases or controls report their exposures differently, or when the information is solicited differently” [45]. Subjects from different inpatient wards of the hospital who had no history in road traffic injuries were enrolled. Inclusion for the control patients was limited to those who were hospitalized in wards other than trauma wards or not hospitalized originally due to an accident. Hospitalization due to injury was considered to be regardless of the mechanism causing injury meaning that any record of intentional or unintentional injury hospitalization such as due to traffic injuries, burn injuries, fall injuries or other injury mechanisms were excluded to be enrolled as controls. Controls were matched by residency in East Azerbaijan province, ability to walk, and age. Exclusion criteria for control patients were having history of hospital admission due to injuries, severe disability and non-ambulatory patients, patients attending the hospital for outpatient visits, and non-residents receiving treatment at the hospital.

### Variables and data collection procedures

The collected information was categorized in three separate data group areas.

First category included background and demographic characteristics such as sex, age, marital status, education level, job, economic status, average walking (distance or time) in daylight and night time, and the type of injuries incurred to pedestrians over accidents. Marital status was defined as married (based on Iranian legislations) and single (including those never married, divorced, widowed and separated). The educational level according to Iranian educational system was defined as an ordinal variable including four categories and were reported along with their equivalents in terms of the International Standard Classification of Education (ISCED) which is a statistical framework for organizing information on education maintained by the United Nations Educational, Scientific and Cultural Organization (UNESCO) [46]. The categories were as follows:

1. Bachelor and master degrees equivalent to the ISCED levels 6 & 7
2. Associate degree (ISCED level 5)
3. Middle school & high school (ISCED levels 2,3 & 4)
4. Illiterate and elementary equivalent to ISCED levels below 2.

For assessing the economic status two indicators were used as household income and self-rated expenditure capacity. Self-rated expenditure capacity is a validated tool assessing the expenditure capacity through six components including clothing expenditure, fruit foods expenditure, other food expenditure, jewelry and prestige expenditure, Traveling expenditure, and education expenditure each measured through a 5-point Likert scale summing to form the “self-rated expenditure capacity” [47, 48]. In current study we had measure the occupation in three major categories including laborers, farmers and other occupations (governmental employees and self-employed). In Iranian health system, until recently, laborers and farmers had their own social welfare and insurance systems. The average walking time for each participant was measured as the surrogate for amount of pedestrian exposure to traffic environment.

Second category included environmental factors, including places and times where injuries occurred. The third category of formation addressed the ADHD symptoms obtained through structured clinical interviews using the screening version of the Conner’s Adult ADHD Rating Scale (CAARS), which assesses ADHD symptoms using 30 questions. It utilizes a 4-point Likert-scaled answer format in which respondents are asked to rate items pertaining to participant’s problems. The validity and reliability of CAARS as a screening instrument is in accordance with DSM-IV guidelines (the construction, structured clinical). Screening included several CAARS subscales as; attention deficit index (subscale A with 9 items); hyperactivity-impulsivity index (subscale B) with 9 items; and ADHD index with 12 exclusive items.

The structured clinical interviews also included questions based on criteria for ADHD derived from the DSM-IV translated from English to Persian with slight adjustments for questions to be understandable and acceptable for the Iranian patients. The self-report screening version of the Conner’s Adult ADHD Rating Scales (CAARS-S:SV) and Observer Screening Version of the Conner’s Adult ADHD Rating Scales (CAARS-O:SV) have earlier been translated and validated in Persian in 2012–2013.

Internal consistency of the Persian version of both the CAARS-S: SV and CAARS-O: SV using Cronbach’s alpha for all four subscales have been within a range of 0.82–0.97. Content validity of the Persian versions of CAARS-S:SV and CAARS-O:SV was confirmed by the adjusted kappa of over 0.76 for all items comprising the CAARS-S:SV and CARRS-O:SV [49].

The questionnaires were completed by the same trained interviewers for cases and controls through face to face interviews in illiterates. People with enough literacy self-administered this scale.

In these cases, questionnaires were checked (and respondents asked for additional information, if needed) by the interviewer. To increase the inter-rating agreement, the interviewers passed a training course; used standardized data collection procedure; the data collection activities were monitored; and the study specific aims were masked as recommended before [50].

The data were collected continuously during May 2012 to April 2013 to reach adequate sample size. The sample size was estimated through the pilot phase, comparing the mean ADHD index scores of 9.7 and 8.3 respectively for cases and controls and corresponding standard deviations of 4.83 and 4.55 respectively. A maximum 5% type 1 and 20% type 2 errors were assumed for power estimation. The sample size calculation was made using SAMPSI function in Stata statistical software package. Cases were matched through frequency matching method. In this kind of matching process, matching is not applied individually and it is conducted for groups of subjects. That is to say a group of controls is matched to a group of cases with respect to a given characteristic such as age or sex in current study. For instance, if in a case-control study with 100 cases where there are 40 males and 60 females, we would select a control group having the same gender distribution. Frequency matching is useful when the distribution of cases for a confounder differs markedly from distribution of that variable in source population which is the case with both sex and age in current project.

### Statistical analysis

Descriptive statistics were provided for background variables separately in case and control groups. For comparison of the association of the risk factors with injury among groups, the independent t and Mann-Whitney tests were used according to the distribution of the data. For the categorical variables, Chi-Square and Fisher Exact tests were used. Crude and adjusted Odds Ratios (ORs) with 95% Confidence Intervals (CI) were reported. To investigate the potential independent predictive role for ADHD scales and exploring other determinants of hospitalization due to pedestrian injuries, multiple binary logistic regression analysis was used. Injury group membership was considered as dependent variable in bivariate and logistic regression analysis. Taking into account the main purpose of the current study and the inherent collinearity between various ADHD subscales, the final multiple regression models were developed using only the A, B and D subscales of the screening tool through three separate models. However, the first model that included D-subscale answers to the main research question of an association between ADHD and pedestrian injuries. D-subscale at same time has the highest sensitivity and specificity in detecting adult ADHD disorder regardless of the disorder subtype (subscales A & B). The data were analyzed using the Stata version 13 statistical software package (Stata Corp. Texas 77,845 USA). *P*-values below 0.05 were considered as statistically significant.

### Ethical issues

The study protocol was approved by the institutional ethics committee in Tabriz University of Medical Sciences. Written informed consent was obtained from all participants after explaining the aim of the study.

## Results

### Background information

Here we present some background information to have a better understanding of the study sample including cases and controls. Pedestrian injury victims, equaling 177 patients, were compared with 177 appropriate controls. Men comprised 86.4% of all the study subjects. Mean age of the participants was 39.7 years with a standard deviation of 14 years. Males comprised 306 (86.4%) of participants and there were 48 (13.6%) females injured. Some demographic characteristics for cases and controls are demonstrated in Table 1.

**Table 1:** Comparison of the demographic characteristics of injured pedestrians (cases) and pedestrians without any injuries (controls), East Azerbaijan province (Iran)

Variables		Cases	Controls	*P*-value
		N. (%) Mean (SD)	N. (%) Mean (SD)	
Sex	Male	153 (50)	153 (50)	1^**^ (Matched)
	Female	24 (50)	24 (50)	
Age	Mean (SD)	40.05 (14.3)	39.3 (13.6)	0.63(Matched)^*^
Marital status	Single	41 (23.2)	39 (22.0)	0.1^*^
	Married	136 (76.8)	138 (78.0)	
Education	Illiterate and elementary (ISCED levels < 2)	89 (50.3)	34 (19.2)	0.001^**^
	Middle school & high school (ISCED levels 2,3 & 4)	68 (38.4)	93 (52.5)	
	Associate degree (ISCED level 5)	15 (8.5)	19 (10.7)	
	Bachelor and master degrees (ISCED levels 6 & 7)	5 (2.8)	31 (17.5)	
Occupation	Farmer	10 (5.6)	9 (5.1)	0.001^*^
	Laborer	39 (22.0)	13 (7.3)	
	Others	128 (72.3)	155 (87.6)	

*: *P-value* comes from independent t-test

**: *P-value* comes from Chi-square test

Mean walking times in day and night were compared for the cases and controls and it was found that the mean walking time was significantly higher for cases than controls both during the day and night (Table 2).

**Table 2:** Comparison of mean daily walking time for cases (pedestrians injured in road traffic accidents), and controls, East Azerbaijan province (Iran)

Variables	Cases	Controls	*P*-value
	Mean (SD)	Mean (SD)	
Mean walking time at day (hours)	2.91 (2.24)	1.86 (1.79)	0.001
Mean walking time at night (hours)	0.31 (0.64)	0.18 (0.47)	0.005*

*P*-value< 0.05

*P-value based on Mann-Whitney test

Hits by motorized vehicles caused 43.50% of victims to be injured at lower extremities and 31.64% of them had more than two organs injured. The majority of accidents (59.32%) had occurred in 2-lane roads. Most accidents (37.30%) occurred between 6 A. M (Ante Meridiem) to 12 P. M (Post Meridiem). Majority of the crashes (80.8%) occurred on weekdays (Saturday-Wednesdays) compared to 19% occurring on weekends.

### Association between pedestrian injuries and its potential correlates

In order to identify potential confounders of study hypothesis to be controlled later through multivariate analysis, using bivariate analysis methods, here we investigated co-variation of study variables with being hospitalized due to pedestrian injuries as the outcome variable. Table 3 demonstrates the findings of simple binary logistic regression analysis reporting crude odds ratios (OR) along with their 95% confidence intervals. Of all the variables investigated in bivariate analysis, education level, average walking time at day, average walking time at night, household income, self-rated expenditure capacity, smoking, ADHD hyperactivity/impulsiveness subscale, and ADHD index scores were associated with pedestrian injuries. ADHD subscale A (attention deficit) had a non-significant *p*-value of 0.08.

**Table 3:** Bivariate associations assessed between pedestrian crash hospitalization and potential variables investigated in case-control study, East Azerbaijan, Iran

Comparison groups	Cases (*n* = 177)	Controls (*n* = 177)	Crude odds ratios (OR)	95% Confidence Interval
Variables	N(%)/ Mean (SD)	N(%)/ Mean (SD)		
Marital status				
Single (RG^a^)	41 (23.2)	39 (22.0)		
Married	136 (76.8)	138 (78.0)	0.93	0.56–1.54
Education level				
Illiterate and elementary (ISCED levels < 2)	89 (50.3)	34 (19.2)		
Middle school & high school (ISCED levels 2,3 & 4)	68 (38.4)	93 (52.5)	0.27	0.16–0.46
Associate degree (ISCED level 5)	15 (8.5)	19 (10.7)	0.30	0.13–0.66
Bachelor and master degrees (ISCED levels 6 & 7)	5 (2.8)	31 (17.5)	0.06	0.02–0.17
Job				
Farmer (RG)	10 (5.6)	9 (5.1)		
Worker	39 (22.0)	13 (7.3)	2.7	0.90–8.09
Self-employed	128 (72.3)	155 (87.6)	0.74	0.29–1.88
Average walking time during day light (hours)	2.91 (2.24)	1.86 (1.79)	1.31	1.16–1.47
Average walking at night (hours)	0.31 (0.64)	0.18 (0.47)	1.57	1.03–2.39
Self-rated expenditure capacity	12.8 (3.5)	9.98 (4.1)	0.82	0.78–0.88
Household income				
< 161USD (RG)	78 (44.3)	28 (15.8)		
161–321 USD	80 (45.5)	88 (49.7)	0.32	0.19–0.55
321–482 USD	12 (6.8)	39 (22.0)	0.11	0.05–0.24
482 > USD	6 (3.4)	22 (12.4)	0.09	0.03–0.26
Age	40.05 (14.21)	39.34 (13.54)	1.003	0.98–1.01
Smoking				
None-smoking (RG)	106 (59.9)	136 (76.8)		
5–9 per days	48 (27.1)	23 (13.0)	2.67	1.53–4.67
10–15 per days	23 (13.0)	18 (10.2)	1.63	0.84–3.19
ADHD subscales^b^				
Attention deficit subscale	5.58 (3.87)	4.87 (3.62)	1.05	0.99–1.11
Hyperactivity-impulsivity subscale	7.83 (3.84)	6.75 (3.58)	1.08	1.02–1.14
ADHD index	9.58 (5.12)	8.43 (4.63)	1.04	1.004–1.09
Psychiatric referral history				
No (RG)	170 (96.0)	168 (94.9)		
Yes	7 (4.0)	9 (5.1)	0.76	0.27–2.11

^a^Reference group for calculating odds ratios

^b^ ADHD subscales: A: DSM-IV inattentive symptoms / B: DSM-IV hyperactivity/ impulsive symptoms / C: DSM-IV ADHD symptoms total / D: DSM-IV ADHD index

### Independent association of ADHD screening score with pedestrian injuries

The main research question is answered in this section showing the independent association of ADHD scale scores with the outcome variable while controlling for potential confounding effects of other cofactors. Three final regression models were developed to answer the research questions including five regressors as independent determinants of pedestrian injuries. Each of the three models included the three ADHD subscales as attention deficit subscale, hyperactivity/impulsiveness subscale and ADHD index score respectively. Table 4 shows the findings of the final multiple binary logistic regression analysis reporting adjusted odds ratios and their 95% Confidence Intervals. The odds ratio for ADHD subscale scores are for one single score increment in each subscale score.

**Table 4:** Adjusted odds ratios for the factors included in final multiple logistic regression analysis of pedestrian injury determinants separately for ADHD subscales in East Azerbaijan, Iran

Model 1: Independent association for D-scale (ADHD Index)						
Predictors	Odds Ratio	Std. Err.	z	P-value	[95% Conf. Interval]	
ADHD Index score	1.06	0.03	2.44	0.02	1.01	1.12
Family income						
< 161USD	Ref. group					
161–321 USD	0.55	0.17	−1.98	0.05	0.30	0.99
321–482 USD	0.30	0.14	−2.58	0.01	0.12	0.75
> 482 USD	0.36	0.22	−1.64	0.10	0.11	1.22
Self-rated expenditure capacity	0.92	0.04	−2.02	0.04	0.85	1.00
Education level						
Illiterate and elementary (ISCED levels < 2)	Ref. group					
Middle school & high school (ISCED levels 2,3 & 4)	0.45	0.13	−2.79	0.01	0.25	0.79
Associate degree (ISCED level 5)	0.68	0.30	−0.87	0.38	0.28	1.63
Bachelor and master degrees (ISCED levels 6 & 7)	0.14	0.08	−3.44	0.00	0.05	0.43
Mean total daily walking at day (hour)	1.15	0.07	2.24	0.03	1.02	1.30
_cons	3.07	1.57	2.21	0.03	1.13	8.34
Model 2: Independent association for attention deficit index						
Predictors	**Odds Ratio**	**Std. Err.**	**z**	**P-value**	**[95% Conf. Interval]**	
Attention deficit subscale score	1.08	0.04	2.17	0.03	1.01	1.16
Family income						
< 161USD	Ref. group					
161–321 USD	0.52	0.16	−2.13	0.03	0.29	0.95
321–482 USD	0.29	0.13	−2.67	0.01	0.12	0.72
> 482 USD	0.38	0.24	−1.53	0.13	0.11	1.30
Self-rated expenditure capacity	0.92	0.04	−1.96	0.05	0.86	1.00
Education level						
Illiterate and elementary (ISCED levels < 2)	Ref. group					
Middle school & high school (ISCED levels 2,3 & 4)	0.45	0.13	−2.77	0.01	0.26	0.79
Associate degree (ISCED level 5)	0.65	0.29	−0.96	0.34	0.27	1.57
Bachelor and master degrees (ISCED levels 6 & 7)	0.14	0.08	−3.49	0.00	0.04	0.42
Mean total daily walking at day (hour)	1.16	0.07	2.41	0.02	1.03	1.31
_cons	3.51	1.74	2.54	0.01	1.33	9.26
Model 3: Independent association for hyperactivity-impulsivity index						
Predictors	**Odds Ratio**	**Std. Err.**	**z**	**P-value**	**[95% Conf. Interval]**	
Hyperactivity-impulsivity subscale score	1.11	0.04	2.91	0.00	1.03	1.18
Family income						
< 161USD	Ref. group					
161–321 USD	0.53	0.16	−2.05	0.04	0.29	0.97
321–482 USD	0.28	0.13	−2.73	0.01	0.11	0.70
> 482 USD	0.31	0.19	−1.88	0.06	0.09	1.05
Self-rated expenditure capacity	0.92	0.04	−1.98	0.05	0.85	1.00
Education level						
Illiterate and elementary (ISCED levels < 2)	Ref. group					
Middle school & high school (ISCED levels 2,3 & 4)	0.46	0.13	−2.71	0.01	0.26	0.81
Associate degree (ISCED level 5)	0.70	0.32	−0.78	0.44	0.29	1.70
Bachelor and master degrees (ISCED levels 6 & 7)	0.16	0.09	−3.24	0.00	0.05	0.49
Mean total daily walking at day (hour)	1.15	0.07	2.31	0.02	1.02	1.31
_cons	2.55	1.33	1.79	0.07	0.92	7.11

As could be found in Table 4, the first model, while controlling for confounding effects of other variables, tested the hypothesis whether adults with a higher adult ADHD screening score (based on ADHD index) have higher likelihood of being injured and hospitalized due to pedestrian injuries. It showed that one single score increment in ADHD index increases the likelihood of being in pedestrian injury group by 6 % in this setting. Model 2 and model 3 tested weather the potential association between being in pedestrian injuries group and ADHD screening scores could vary with respect to the two types of attention deficit and hyperactivity/impulsiveness symptoms or not. The results showed that although both ADHD symptom types were associated with higher likelihood of being in pedestrian injuries group, hyperactivity/impulsiveness symptoms’ score had a minimally stronger role than attention deficit symptoms.

Injury group membership was considered as dependent variable logistic regression analysis.

## Discussion

### Main findings

The present study revealed that pedestrians who had road traffic injuries leading to hospitalization, were more likely to have higher scores on ADHD symptoms. As about 40% of mortalities due to RTAs in East Azerbaijan (the study setting) occur either at the crash scene or on the way to the hospital, it was not possible to include these victims due to lack of the interview potential. We didn’t find any publication to report an association between ADHD and severity of RTAs leading to higher pre-hospital death proportion among crash victims with ADHD than those without it. If no such an association is assumed, our results may also have some generalizability to RTAs regardless of injury severity. Otherwise, if we assume that people with ADHD are either more or less likely to have injuries with higher severity than victims without the disorder, an under-estimation or over-estimation of the odds ratio of ADHD scores in our cases may be expected for extrapolation of results further than hospitalized pedestrian injuries.

### ADHD and pedestrians crash injuries

The three most important types of injured road users in traffic environment that may be affected by symptoms of ADHD include; drivers (mainly car drivers, bus drivers and cargo-vehicle drivers), riders (motorcycle and bicycle riders) and pedestrians. The current study is among the very few studies that have specifically investigated an association between ADHD symptoms and risk of pedestrian crash. The association between pedestrian injuries and ADHD is largely ignored in literature and we retrieved one article specifically investigating the ADHD in pedestrian safety as well as two other simulator studies [51–54]. The study conducted in virtual reality interestingly found that adolescents with ADHD had greater variability in road-crossing behavior, and showed twice as many collisions as compared to controls (**52**). As measured by Conner’s adult ADHD screening D-index scores, the present study revealed that pedestrians who had road traffic injuries leading to hospitalization, were more likely to have higher scores on ADHD symptoms.. ADHD may affect the pedestrian crash risk by various mechanisms related to the two main features of ADHD i.e. attention deficit and hyperactivity/impulsiveness symptoms. The psychodynamic approach, the cognitive approach, and the character and human motivation approach are earlier discussed as explanations for risky behavior among young people. The sensation-seeking theory is reported to be the most relevant and plausible link with ADHD and risk-taking motorcycle riding behaviors [30, 55]. This may also apply to some extent for pedestrian unsafe risk-taking behaviors either. In case of pedestrians, many risk-taking behaviors may happen when cross-walking freeways. Moreover, ADHD patients may make hazardous decisions about vehicle approach times due to their perceptual difficulties in judging the time-to-arrival of approaching vehicles. The time perception impairment and executive function deficits among those with ADHD may explain their problems in using the available gap to cross safely between vehicles [56].

### ADHD comorbidities and pedestrian crash injury

The higher likelihood of traffic injuries in pedestrians with ADHD, if also confirmed through future cohort studies, may be explained through distraction not just due to the main attention deficit symptoms of ADHD. For instance, it has been shown that people with ADHD are more likely to use cell phones for a longer time [57], may be rooted in their talkative manner of communications. This could be an extra source of distraction among pedestrians with ADHD. Moreover, other outcomes of ADHD, such as sleep disorders, substance misuse, and problems at workplace that may affect pedestrians awareness while crossing a road or walking in traffic environment [8, 58, 59]. An interesting study on risk factors for adverse driving outcomes in Dutch adults with ADHD demonstrated that alcohol use, and high levels of anxiety and hostility are highly prevalent among adults with ADHD interestingly discussing the mediating role of them on the risk for negative driving outcomes in drivers with ADHD [17]. This reveals that not all our explanation of the association of ADHD and traffic injuries should be put on direct effect of ADHD symptoms. Moreover, considering results of a recent review on ADHD and relative risk of accidents in road traffic [26] that showed an overestimation of risk may occur if the with comorbid ODD and/or CD is not taken into account, We recommend future research to consider parallel assessments of scale-based scores along with psychiatric diagnosis of ADHD and its comorbidities among pedestrians either.

### Simulation studies and ADHD

Although, evidence on adult pedestrian safe walking behavior in subjects with ADHD is quite scarce, simulation study on children playing the pedestrian role in simulation environment showed that children with combined type ADHD, prefer to choose riskier environments to cross within, the shorter amount of time left to spare upon reaching the other end of the cross walk, and the increased frequency of hits/close calls they experienced [52]. Professor Barkley recites in a commentary on the study by Nikolas et al. that the specific cognitive-behavioral risk mechanisms are likely part of the larger domain of executive dysfunctioning and delay aversion inherent in ADHD [60].

### Socio economic status, ADHD and crash injury

In our study, individuals with lower economic status, as assessed by household income and self-rated expenditure capacity, were more involved in road traffic accidents leading to hospitalization than controls. Household income and the primary carer’s education level are shown to be inversely related with child pedestrian injury risks [61]. Although economic status may be associated with both with ADHD and RTAs, our results showed that ADHD screening score is a determinant of pedestrian injuries independent of the economic status.

Implications: We explored the association of ADHD score with pedestrian injuries in a given population in Iran. Due to paucity of information in this area, we don’t exactly know whether the study setting can modify the effect of ADHD scoring on such an association assuming that the whole picture of traffic behaviors including drivers, riders and pedestrians as well as the infrastructural safety may affect the crash risk and interact with its determinants including ADHD. At the same time the results based on association of ADHD symptom scores with traffic injuries puts forward a hypothesis that those with higher symptom scores even if not diagnosed to have ADHD may have higher likelihood of injuries than those with lower scores. How severe injuries might have impacted cognitive function that affects the assessment for ADHD symptoms is a concern that we raise in the current study. Two aspects of this could be considered. First is that head injuries may also be an etiology (reverse causality) and secondly the information bias that may be caused by impaired cognitive function. These should be interpreted accordingly. This could be reliably addressed in cohort studies that measure about ADHD along with other pedestrian injury risk factors. Such cohorts are quite rare worldwide. Recently the Persian Traffic Cohort which is a population-based road safety specific population cohort (https://safety.tbzmed.ac.ir/page/54/PREC.html) has been started and fortunately has included ADHD related measurements. However, it may take half a decade to gain accurate data from such cohorts and we need to consider the evidence from currently available studies. The present study used a case-control design. Like all other case-control studies, the potential for various biases should always be taken into account. The most important type of bias needing to be minimized is selection bias. “Selection bias exists when cases or controls are selected in a way that is not representative of the respective exposure distributions in the study base.” [45]. To minimize the selection bias is to ensure that the cases and controls come from the same study base which is called a secondary study base in case of hospital-based case-control studies. Recommendations has been given by Wacholder in this regard that were considered in developing the present study as stated earlier in methods [44]. Recall bias which is a known information bias in case-control studies “may occur if cases aware of the hypothesis tend to report their exposures more fully than controls, with resultant overestimation of the odds ratio” [45]. A lack of awareness of the hypothesis diminishes the chance of information bias and adding this to use of hospital controls versus population controls, as in present study, would be promising in this regard. However, there is no guarantee for full control of bias in any case-control study and caution should always be taken in interpreting the results of these types of studies.

After adequate experimental research, among pedestrians who are in traffic accidents and suffer high ADHD screening scores, ADHD treatments (medication and EF skills training) may be an effective way to reduce accidents. The current case-control study was planned based on mechanism of injury not the type of injury. There have been several studies conducted focousing on type of injury, such as head injuries or burn injuries, rather than mechanism of injury such as traffic injuries or fall injuries. Actually there seems to be more plausible for ADHD to affect specific mechanisms of injuries rather than specific types of injuries. It may be interesting for future research to consider parallel assessment of this issue. Although, in case of pedestrian injuries it may not be a major problem, it should be appropriately addressed with respect to motorcycle driver injuries. This is because, the pattern of injuries among motorcyclists is substantially different from other traffic accidents and at the same time choosing to travel with a motorcycle may be considered in some cases as a risk taking behavior or determined by socioeconomic level both of which are associated with having ADHD. However, being a pedestrian is a common traffic role that nearly everyone with a normal life owns.

### Limitations

In present study, our methodology didn’t permit us and we didn’t aim to prove a causal association of clinically diagnosed ADHD and pedestrian injuries. Instead, we investigated a potential predictive role for ADHD scores based on self-reporting. This was the reason we, unlike some similar previous studies, avoided using terminologies such ad risk factors or causal relationship neither in our findings or conclusions. We just tried to discuss the plausibility of such an association for screening purposes and hypothesis generation for future studies. However, findings of present research, as presumably the first non-simulation case-control study specifically on adult ADHD symptoms and pedestrian injuries, puts forward implications for future research on parallel use of clinical diagnosis and self-report scales. Additional limitations that could also be noted include the lack of a full ADHD diagnosis along with the collateral (other) reports of ADHD symptoms. One more limitation of this study is that the authors did not exclude based on diagnoses or medications known to have impact on executive functions or cognitive control.

## Conclusions

Adult ADHD screening score can predict pedestrian injuries leading to hospitalization independently from sex, age, economic status, educational level and pedestrian exposure to traffic environment (average walking time). This can provide hints towards pedestrian safety promotion in public health and clinical practice as well as implication for designing future research. For instance, surgeons noticing some symptoms among injured pedestrians should consider an ADHD screening or psychological consultation. Similarly, psychiatrists visiting patients with adult ADHD should inform them about potential risks of pedestrian injuries. Adult ADHD screening may be considered at research or policy levels for given target groups such as relatives of children with ADHD.

## Data Availability

The datasets used and/or analysed during the current study are available from the corresponding author on reasonable request.

## Abbreviations

ADHD: Attention Deficit Hyperactivity Disorder
CAARS-S:SV: Conner’s Adult ADHD Rating Scales the Self-report Screening Version
ISCED: International Standard Classification of Education
RTA: Road Traffic Accidents
SD: Standard Deviation
RG: Reference Group
EF: Executive Function
